# The contribution of advisory committees and public involvement to large studies: case study

**DOI:** 10.1186/1472-6963-10-323

**Published:** 2010-12-02

**Authors:** Mike Slade, Victoria Bird, Ruth Chandler, Jo Fox, John Larsen, Jerry Tew, Mary Leamy

**Affiliations:** 1 Health Service and Population Research Department, King's College London, Institute of Psychiatry, Denmark Hill, London SE5 8AF, UK; 2 Service User and Carer Involvement Coordinator in Research and Development, Sussex Partnership NHS Foundation Trust, Portsmouth, UK; 3 Department of Social and Social Policy, Anglia Ruskin University, East Road, Cambridge CB1 1PT, UK; 4 Rethink, 89 Albert Embankment, London SE1 7TP, UK; 5 Institute of Applied Social Studies, University of Birmingham, Edgbaston, Birmingham B15 2TT, UK

## Abstract

**Background:**

Many large studies have complex advisory committee structures, yet there is no empirical evidence regarding their optimal composition, scope and contribution. The aim of this study was to inform the committee and advice infrastructure for future research studies.

**Methods:**

In the context of a five-year study funded by the UK National Institute for Health Research, three advisory committees were formed. In addition, advice was obtained from individual experts. All recommendations received in the start-up phase (first seven months) of the study were recorded, along with the decision about implementation of the recommendation. A particular focus was on the impact of public involvement.

**Results:**

A total of 172 recommendations were made, including 70 from 20 individual experts. The recommendations were grouped into five emergent themes: Scientific, Pragmatic, Resources, Committee and Collaboration. Most recommendations related to strengthening existing components or adding new components to the study protocol. Very few recommendations either proposed removing study components or contradicted other recommendations. Three 'implementation criteria' were identified: scientific value, pragmatic feasibility, and paradigmatic consistency. 103 (60%) of recommendations were implemented and 25 (15%) were not implemented. The benefits identified by the research team were improved quality and confidence, and the costs were increased cognitive demands, protocol revision time, and slower progress.

**Conclusions:**

The findings are discussed in the context of the wider literature on public involvement in research. Six recommendations are identified. First, have a clear rationale for each advisory committee expressed as terms of reference, and consider the best balance between committees and individual consultation with experts. Second, an early concern of committees is inter-committee communication, so consider cross-representation and copying minutes between committees. Third, match the scope of advisory committees to the study, with a less complex advisory structure for studies with more finalised designs. Fourth, public involvement has a mixed impact, and relies on relationships of trust, which take time to develop. Fifth, carefully consider the match between the scientific paradigm applied in the study and the contribution of different types of knowledge and expertise, and how this will impact on possibilities for taking on advice. Finally, responding to recommendations uses up research team resources, and the costs can be reduced by using the three implementation criteria.

## Background

The national research strategy for the National Health Service (NHS) in England identifies a goal of "*establishing the NHS as an internationally recognised centre of research excellence*" [1]. Approaches outlined in this strategy include increasing participation in health research with high-quality protocols, with a particular focus on increasing the number of people who enter multi-centre trials. One new mechanism is the introduction of Programme Grants for Applied Research, described as "*substantial, prestigious awards...over three- to five-year periods, in order to...fund a series of related projects which form a coherent theme in an area of priority or need for the NHS*" (p. 25).

The current study was undertaken in the context of a larger research programme. In November 2008 a £2 m (US$3.2 m) five-year (2009-2014) Programme Grant was awarded for a study titled "REFOCUS: Developing a recovery focus in adult mental health services in England". REFOCUS comprises a series of inter-linked sub-studies including systematic reviews and meta-analytic studies, qualitative semi-structured interviews and focus groups, national surveys, consensus methods, outcome measure development and psychometric evaluation. These lead to a multi-centre cluster randomised controlled trial of a complex intervention, supported by process evaluation and innovation in clinical end-point assessment. Three of the study researchers are undertaking PhDs related to the study. More detailed information on REFOCUS is available at researchintorecovery.com.

The start-up phase of any study is crucial, since many key design and implementation decisions are evaluated and finalised. This phase may be especially important in relation to large research programmes with multiple components, due to their added complexity. Clear guidance exists in relation to oversight committees for studies, including appropriate arrangements for trials to be monitored by a Trial Steering Committee [2]. Some components of these oversight arrangements have been investigated, such as the role of data monitoring and interim analysis in the DAMOCLES Study [3]. However, the role of advisory committees - which provide advice and expertise to support the study but without a formal oversight function - has not been investigated. Although it is common to form steering committees to inform research studies, we could locate no empirical evidence regarding the optimal composition and timing of advisory committees, or the nature and impact of their contributions. The aim of the study reported here was to begin this process, by characterising and evaluating the contribution of various forms of expert input to REFOCUS during the start-up phase, by collecting data during the first seven months of the study. A particular emphasis was on the impact of public involvement, in this case the advisory contributions made from mental health service users and carers. There is an evidence gap on how public involvement improves the quality and relevance of research, since public involvement is "*by its nature a complex, social *process" [4] (p. 92). Assessing the impact of this involvement is difficult, and sometimes impossible [5]. The overall goal was to inform the committee and advice infrastructure for future research studies.

## Methods

### Design

Case study of contributions from committees and individual experts to one research study.

### Procedure

The initial advisory infrastructure for REFOCUS comprised three committees. The purpose of each committee was described in its terms of reference. First, the Steering Group was composed of the 12 applicants and 7 collaborators named in the proposal. The applicants were academic, clinical researchers, user-researchers or service leads who together provided cross-cutting expertise relevant to the overall aims of the Programme. Collaborators brought methodological expertise relevant to specific sub-studies. The Steering Group was chaired by the Principal Investigator, and the terms of reference were to provide methodological and implementation expertise to contribute to the success of the study. The committee met in May and September 2009. As part of the Steering Group, a cultural and social perspectives sub-group was formed from the Steering Group membership in May 2009, which communicated by email.

Second, the Lived Experience Advisory Panel (LEAP) comprised eight experts by experience, *i.e. *as mental health service users and carers. Recovery ideas have emerged from people with personal or 'lived' experience of mental illness [6,7], but the mental health system has been criticised for co-opting or commandeering the recovery approach [8,9]. The purpose of LEAP was to retain the integrity of these recovery values, and the terms of reference were therefore to be a 'critical friend' to REFOCUS: bringing the perspective of lived experience to the methodological and implementation decisions. The LEAP committee was chaired by a voluntary sector representative, and met in May and September 2009.

Finally, the International Advisory Board (IAB) comprised nine experts from Australia, England, Ireland, Scotland and the USA. The terms of reference for the IAB were to provide an international perspective both on research evidence and best recovery-related practice in mental health services. IAB meetings were chaired by the Principal Investigator, and one meeting was held in May 2009.

Additionally, specific experts were identified and consulted with about specific issues. These experts included both members (n = 9) and non-members (n = 11) of advisory committees, and brought the same range of expertise as the applicants described earlier. We refer to the three advisory committees and the additional experts collectively as 'advisors'. The REFOCUS Study began in May 2009, and recommendations made by advisors were recorded until November 2009. All three committees will however continue to operate until the end of the study, with the IAB meeting annually and the Steering Group and LEAP meetings biannually.

A recommendation was defined as a course of action that was recommended by an expert or a committee as advisable, and included both cognitive (i.e. to think about something) and behavioural (i.e. to do something) recommendations. All recommendations were recorded. Recommendations made in committee meetings or afterwards by email as part of a committee follow-up discussion were attributed to the committee. Recommendations made in individual meetings or afterwards by email were attributed to the individual expert (even when they also were members of an advisory committee but were contributing a recommendation outside of a committee follow-up discussion thread). Each recommendation was then discussed by the research team, and a decision about implementation was recorded, comprising either Implemented, Not implemented or Undecided (when the recommendation related to a later phase of the study, such as a reference to include in a resulting publication). The rationale for the implementation decision was also recorded. The content of recommendations was analysed to identify emergent themes and sub-themes.

## Results

Advisors made 172 recommendations to inform the study between May 2009 and November 2009. These comprised 37 from IAB, 20 from LEAP, 36 from the Steering Group, 9 from the social and cultural sub-group, and 70 from 20 individual experts. The experts comprised two IAB members (making 5 recommendations), seven Steering Group members (making 42 recommendations), and 11 non-members (making 23 recommendations).

Although there was some duplication of recommendations from different sources, there was no clear pattern of overlap. The 172 recommendations were grouped into five emergent themes, each with sub-themes, shown in Table 1.

**Table 1:** Recommendations made by advisory committees and experts

Recommendation theme	Sub-theme	**IAB**^**1**^	**SG**^**2**^	**LEAP**^**3**^	**SCS-G**^**3**^	Individual experts	Total
**1. Scientific**		**15**	**21**	**12**	**9**	**45**	**102**
	*Design advice*	11	14	8	7	33	73
	*Suggestion for a study extension*	0	4	1	0	3	8
	*Recommendation for intervention content*	3	1	1	0	4	9
	*Conceptual suggestion*	0	1	2	2	4	9
	*Advice specific to PhDs*	1	1	0	0	1	3

**2. Pragmatic**		**6**	**1**	**1**	**0**	**2**	**10**
	*Implementation advice*	2	1	0	0	2	5
	*Recommendation for language to use*	1	0	1	0	0	2
	*Advice about dissemination*	3	0	0	0	0	3

**3. Resources**		**11**	**8**	**0**	**0**	**19**	**38**
	*Published work to consider*	6	1	0	0	3	11
	*Unpublished work to consider*	3	5	0	0	3	11
	*Expert person/group to contact*	2	2	0	0	13	17

**4. Collaboration**	*Ongoing contact about specific issue*	**0**	**0**	**0**	**0**	**4**	**4**

**5. Committee**	*Role and composition of committee*	**5**	**6**	**7**	**0**	**0**	**18**

**Total**		**37**	**36**	**20**	**9**	**70**	**172**

^1^International Advisory Board. ^2^Steering Group. ^3^Lived Experience Advisory Panel. ^4^Social and Cultural sub-group.

Examples of Scientific recommendations included accessing an existing database (Design advice), involving carers (Study extension), using video clips as a training aid (Recommendation for intervention content), and to consider the cyclical nature of recovery (Conceptual suggestion). Examples of Pragmatic recommendations included liaising closely with trial sites (Implementation advice), revising the term 'manual' (Recommendation for language to use) and providing online information about the study (Advice about dissemination). The Resources recommendations identified publications or other experts to access. Examples of Committee recommendations included composition, terms of reference, cross-committee representation and circulation of minutes, and clarity about role in decision-making in REFOCUS. An example of a Collaboration recommendation was the request by an expert to liaise in the future about a Department of Health initiative on personalisation. Most recommendations were either strengthening existing components or adding new components to the study protocol. Very few recommendations either proposed removing study components or contradicted other recommendations.

The criteria for implementing recommendations were not specified in advance, but discussions about implementation were observed by the research team to involve three implementation criteria: scientific value; pragmatic value; and paradigmatic consistency. One reason not to implement the recommendations was where it added little or no **scientific **value, such as involving a new advisor with the same expertise as an existing advisor. The **pragmatic **criterion related to recommendations which did strengthen the current design but with unmeetable cost (in time and money) implications for the study, such as developing a new focus on social capital interventions. Finally, recommendations were sometimes not implemented on **paradigmatic **grounds when the suggestion was reasonable from within an alternative frame of reference but incompatible with the goal of contributing evidence within the evidence-based mental health paradigm [10]. For example, the term 'manual' (for the randomised controlled trial intervention) was criticised since pro-recovery working is individualised, but retained as the standard term used in clinical practice. Overall, for recommendations to be implemented they had to add scientific value, be viable within the study resources, and be consistent with the overall scientific framework being used.

The decision about implementation for these 172 recommendations is shown in Table 2. These are shown in month order, to make visible any changes over time.

**Table 2:** Implementation of recommendations from advisory committee and experts

Recommendation	Month 1	Month 2	Month 3	Month 4	Month 5	Month 6	Month 7	Total
**1. Scientific**	*n = 44*	*n = 0*	*n = 8*	*n = 20*	*n = 19*	*n = 8*	*n = 3*	*n = 102*
*Implemented*	36	0	2	10	4	1	3	56
*Not implemented*	8	0	1	3	3	0	0	15
*Undecided*	0	0	5	7	12	7	0	31

**2. Pragmatic**	*n = 8*	*n = 0*	*n = 0*	*n = 2*	*n = 0*	*n = 0*	*n = 0*	*n = 10*
*Implemented*	5	0	0	0	0	0	0	5
*Not implemented*	3	0	0	0	0	0	0	3
*Undecided*	0	0	0	2	0	0	0	2

**3. Resources**	*n = 18*	*n = 0*	*n = 9*	*n = 4*	*n = 6*	*n = 0*	*n = 1*	*n = 38*
*Implemented*	16	0	3	1	2	0	1	23
*Not implemented*	2	0	2	0	1	0	0	5
*Undecided*	0	0	4	3	3	0	0	10

**4. Collaboration**	*n = 0*	*n = 0*	*n = 2*	*n = 0*	*n = 0*	*n = 1*	*n = 1*	*n = 4*
*Implemented*	0	0	1	0	0	1	1	3
*Not implemented*	0	0	0	0	0	0	0	0
*Undecided*	0	0	1	0	0	0	0	1

**5. Committee**	*n = 18*	*n = 0*	*n = 0*	*n = 0*	*n = 0*	*n = 0*	*n = 0*	*n = 18*
*Implemented*	16	0	0	0	0	0	0	16
*Not implemented*	2	0	0	0	0	0	0	2
*Undecided*	0	0	0	0	0	0	0	0

**Total**	*n = 88*	*n = 0*	*n = 19*	*n = 26*	*n = 25*	*n = 9*	*n = 5*	*n = 172*
*Implemented*	73	0	6	11	6	2	5	103
*Not implemented*	15	0	3	3	4	0	0	25
*Undecided*	0	0	10	12	15	7	0	44

Most recommendations from committees were implemented. For IAB, 6 (16%) recommendations were not implemented, comprising 2 Scientific, 2 Pragmatic, 1 Committee and 1 Resources. For LEAP, 4 (20%) were not implemented (3 Scientific, 1 Pragmatic) and 3 (12%) were undecided (3 Scientific). For the Steering Group, 5 (14%) were not implemented (3 Scientific, 1 Resources, 1 Committee) and 6 (17%) were undecided (6 Scientific). For the social and cultural sub-group, 1 (11%) Scientific recommendation was not implemented. Finally, 9 (13%) recommendations from experts were not implemented (6 Scientific, 3 Resources) and 35 (50%) undecided (22 Scientific, 10 Resources, 2 Pragmatic, 1 Collaboration). The non-implementation proportion therefore range from 11% to 20% across the different sources of advice.

The recommendations specifically relating to committees covered amendments to the terms of reference, new members to invite, frequency of meetings, copying minutes between committees, joint meetings and cross-committee representatives, and the role and impact of public involvement.

The research team identified two benefits from the advice. First, the overall research quality was (in the judgement of the research team) improved, by considering a broader range of issues. Second, there was greater confidence that the research was both internationally innovative and of a high methodological quality. The costs identified by the research team (in addition to the financial costs in convening the committees) were the increased demands on researchers to think through and come to a view about each issue raised, the time taken to revise the study protocol, and the consequent slower progress in relation to study deadlines.

## Discussion

This observational study found three main results. First, advisory committees and experts contributed to the study in four ways: scientific advice relating to design questions; pragmatic advice relating to maximising the likelihood of successful implementation and dissemination; providing links to wider resources; and offering opportunities for collaboration. A fifth category of recommendations, on the composition and processes of the advisory committees, did not contribute to REFOCUS and accounted for 10% of the overall recommendations.

Second, 103 (60%) of the 172 recommendations were implemented in order to improve scientific quality. The wide range of recommendations presented challenges to the research team. Three preliminary categories for 'implementation criteria' were identified: scientific (is it valuable?); pragmatic (is it possible?); and paradigmatic (is it consistent?).

Third, it was helpful to record non-implemented recommendations as a means of informing future research. For example, as we discuss in the next section, LEAP recommended a research focus on carers, which was not implemented. Making this decision visible ensures it is both more amenable to debate within the study and can inform future research planning.

To our knowledge, this is the first study to attempt to evaluate the contribution of advisory committees and experts to a large health study. Its primary contribution is therefore in generating hypotheses and preliminary guidance for support structures for future research studies.

### Public involvement

The focus of the research was transforming mental health services towards placing more importance on the expertise by experience of service users and carers. Therefore, the meaningful involvement of 'lived experience' - the perspective of mental health service users or carers - in the study was an important value. All advisory committees contained people with lived experience, but input from this perspective was obtained specifically through LEAP.

Responding to LEAP recommendations was at times challenging. In line with existing research on Public Involvement [4], the overall impact on the research and researchers was mixed. Several positive impacts have been identified in previous studies, which were also evident in our study. The study design was improved through a closer match with the community's needs and interests [11]. The LEAP made the case for culture and ethnicity to be a focus of the research, and this was implemented through extending the study in a number of ways. The phrasing of questions was improved [12]. LEAP identified that the planned approach to goal-setting should be altered to allow people to change their minds over time about their personal goals. The input from LEAP also provided less tangible benefits, including bringing energy and a sense of the study mattering [13], fulfilling its role in being a critical friend by enhancing awareness of implicit beliefs [14], and overall making the process of the study more "*satisfying, even enjoyable*" [15] (p. 14). A final intangible benefit for the study was to challenge the idea that traditional methodologies could be imported without problem to researching recovery. Recovery-oriented research requires the capacity to hold difference between perspectives as part of its process [16], in order to avoiding over-simplified and formulaic conclusions about the complex spectrum of recovery. In the study, these principles had to be balanced against the scientific requirements to remain pragmatic about what can be measured.

Some negative impacts found in other studies did not feature in this study. The issue of control over the research has been noted by others as a negative [17], although we found that careful drafting of terms of reference and the existence of positive pre-existing relationships greatly reduced this issue. Trust was built through all parties demonstrating the willingness can capacity to listen and respond to concerns raised. Public involvement challenges researchers' values and assumptions [18], but in this study that was an intended goal and hence experienced as a positive rather than negative experience.

The primary costs to the research team were "*Higher demands on resources and a slower pace of research*" [4] (p. 65). The need to invest time, energy and money into supporting public involvement is well-documented [19,20]. For example, the involvement of carers as research participants was advocated by LEAP, and initially implemented by the research team (with time spent drafting information sheets and consent forms, and amending the protocol), before it became clear that the resources needed to obtain high quality empirical evidence would be disproportionate. This experience, consistent with other studies [21], points to the need for focussed research about carers and recovery. The absence of a guiding theoretical framework for synthesising multiple perspectives may have contributed to the burden for the research team in responding to LEAP recommendations.

Although not a primary focus of this study, the experience from the perspective of LEAP membership was also considered, and tensions were evident. Service users and carers were asked to be involved in a research study that sought to use quantitative methods, including a randomised controlled trial, to investigate the experience of recovery. This caused ambivalence for people asked to join LEAP and to identify with REFOCUS, and some felt that this approach to recovery - framing deeply personal experiences in quantitative terms - alienated them from the recovery approach and the user values to which they aspired. Lived experience presents a different kind of value and a different kind of knowledge to that of 'service led' knowledge [22], and questions were raised whether insights arising from lived experience were compatible with the positivist scientific paradigm underpinning the research design [23]. Incorporating lived experience perspectives often means that service users invest emotional commitment to research, which lends a different perspective to this process. For example, evaluation of research by service users will have a strong ethical investment, rather than solely being based on positivist scientific principles of methodological soundness. This underpinned much of the ambivalence about being identified with the study. Counter-balancing this ambivalence, there was also recognition that bringing lived experience into dialogue with the study offered the potential to bring a different kind of knowledge. Another motivator for involvement was the desire for service delivery in the NHS to become focussed on supporting individual recovery pathways. For those who did choose to be involved, LEAP members reported that REFOCUS sought to value their contribution and listen to their concerns, which validated their involvement. One mechanism to address concerns about tokenism was to record recommendations and actions taken in order to map the impact and influence of involvement (thus producing the data on which this paper is based).

### Limitations and future research

The study has at least five limitations. First, there was no empirically-based guidance to inform the advisory committee structure. The goal was to balance advice from people with different forms of experience and expertise, but this may not be optimal. It is plausible, for example, that complex aspects such as lived experience require more people to improve the relevance of the research than do less contested areas such as specific methodologies. Our development of a preliminary taxonomy of types of contribution can inform future empirically-based decision-making about the composition and structure of committees, including its membership and its chair. For example, the PI for the study chaired both the IAB and the steering group. This had the advantages of allowing a consistent style of chairing and ensuring that sensitive topics (e.g. about contentious issues or due to local politics) were responded to appropriately, but the PI's professional orientation (*e.g. *clinical background, methodological preferences) may have discouraged particular types of recommendations.

Second, coding recommendations was problematic. It was often difficult to differentiate between comments and recommendations. The consensus within a committee about the recommendation was not recorded, and nor was the strength of the recommendation. The context of the recommendation was not recorded, so the complexity of discussion was not captured. There was no reliability or validity testing (*e.g. *using double rating) for recording and coding recommendations. The methodological rigour could be strengthened in a number of ways. First, by the use of more structured approaches to collecting and analysing the data. This could involve recording and transcribing the committee meetings, and then using a content analysis approach. For example, the use of conversation analysis would provide information about *how *recommendations come to be made [24]. Second, methodology being developed by the "Evidence into Recommendations" group for Guideline Development Groups (GDGs) could be incorporated [25]. They propose a methodology to inform a framework analysis to describe and explain incidents within GDG meetings. Finally, the observational data could be augmented with semi-structured interviews with key stake-holders and focus groups to identify implicit beliefs and processes.

Third, the process of deciding whether to implement recommendations was not evaluated. Our categorisation of recommendation themes and identification of three implementation criteria (scientific value, pragmatic feasibility, paradigmatic consistency) needs further operationalisation, and is based on data collected for only one study so needs replication in other studies, but these categories do provide a preliminary organising framework to facilitate research.

Fourth, the optimal timing of committee composition is unclear. In this study the focus has been on post-funding recommendations from advisory committees, and consistent with standard practice, these committees were formed once the study was funded. There is evidence of scientific benefits from public involvement earlier in the process, when planning the overall design and writing the research proposal [4]. Earlier involvement from LEAP at the design and proposal stage would have strengthened the REFOCUS Study, and reduced the ambivalence about post-funding involvement expressed by some lived experience researchers. This may of course reduce the ability to evaluate the impact of public involvement on the study. It is also plausible, though untested, that the earlier composition of advisory committees more generally (i.e. not just those supporting public involvement) may add value. Earlier involvement from the individual experts would have reduced the number of post-funding recommendations, although this needs to be set against the increased capacity of experts to give time to a funded than a pre-funded study.

The final step towards an evidence-informed advisory committee composition will be an evaluation of benefits and costs which advisory committees bring to a study. Finding the optimal balance between feasibility and obtaining ongoing feedback from diverse perspectives is problematic. It may not be feasible to use an experimental approach (such as random allocation of committee types) to investigate the impact of committee structures on large studies, since the challenges for each study are complex and idiosyncratic. In particular, relationships matter [26,27] - and the nature of the relationships and the demonstrated willingness and capacity to listen may be difficult to allocate to intervention sites. However, it may be possible to rate the methodological quality of study protocols before and after advisory committees have contributed. This would provide some evidence of the benefit accruing from advisors. Furthermore, positive outcomes could be expected in terms of research quality (measured by citation and impact factor metrics) and impact (measured by influence on policy and practice) [28]. Our study suggests that the costs to the research team include time organising and attending committees and responding to post-committee correspondence, cognitive demands in responding to recommendations, and emotional demands of managing relationships between the study team and diverse stake-holders. All of these types of cost are amenable to measurement.

More generally, it is clear from the implementation science literature that several translational blocks can occur between the development of research evidence and practice change [29]. Research is now providing a conceptual framework to understand these 'translational gaps' [30] and identifying approaches to improving implementation [31]. The analogous challenge is addressing 'advice gaps' between experts and the research team. Scientific enquiry into the optimal composition and scope of advisory committees will involve the development of taxonomies of types of recommendations and reasons for implementation/non-implementation (a goal to which this study contributes), and then observational and experimental studies to understand how to maximise the benefits and minimise the costs of responding to advice.

## Conclusions

On the basis of our findings, we can make six recommendations for other large studies.

First, have a clear rationale for each advisory committee. The rationale is best expressed as an agreed terms of reference, and the discussion leading to agreement can both help the committee to form and provide guidance to shape future input. The terms of reference can be informed by the five categories of contribution we have identified. Consider carefully the balance between involving experts through committee membership and through individual consultation, whilst noting that committee members can also be consulted individually.

Second, an early concern of committees is inter-committee communication. Our experience suggests that copying of minutes between each committee should be the norm, and that each committee should be offered the opportunity to have representatives on the other committees. Representation is both beneficial for the study and experienced positively by the representatives.

Third, match the scope of advisory committees to the study. We found that recommendations were adding value, and did not in general contradict each other. This suggests a comprehensive advisory committee structure will improve the quality of decision-making for studies which include a start-up phase during which final design decisions or piloting are undertaken. However, for studies which have a relatively finalised design and need to move rapidly to data collection, too much post-funding advice can be a hindrance to progress, so the advisory committee structure should be reduced at this stage (while their value in the early design phase would be higher).

Fourth, public involvement has a mixed impact. In our study, which had a start-up phase, the impact was on balance very positive. The main cost was time and progress, and the importance of this cost would increase in studies with less start-up time.

Fifth, carefully consider the match between the scientific paradigm applied in the study and the contribution of different types of knowledge and expertise, and how this will impact on possibilities for taking on advice. Although there may not be an easy fit between for example a positivist approach and knowledge arising from lived experience, it may still be possible to take important learning through careful facilitation and mutual willingness to listen and learn. A concrete approach to valuing different forms of knowledge is to acknowledge and record the recommendations and areas of scientific enquiry which, although potentially desirable, cannot be addressed in the study.

Finally, responding to recommendations used up research team resources. The burden on the research team in responding to recommendations should be minimised. The three decision-making criteria which evolved over time were scientific, pragmatic and paradigmatic. Using these criteria in other studies may reduce the cognitive and time costs on the research time in responding to recommendations.

In conclusion, a complex health service research study can require a large network of collaborators. This study has shown that advisory committees can add scientific value and maintain research integrity, has developed a preliminary taxonomy for the types of contributions they make, and has identified that there are also costs involved for the research team. The optimal balance of advice is unknown. On the one hand, the wide range of advice has improved quality and relevance. On the other, excessive consultative input can become unhelpful if it inadvertently places too much pressure on the time and resources of the research team to respond to it. The tipping point between too little and too much advice is unclear. Our finding that recommendations should be implemented when they are valuable, possible and consistent provides a potential framework for future investigation. This study is a contribution to an area of research management which is both important and under-researched.

## Competing interests

The authors declare that they have no competing interests.

## Authors' contributions

MS conceived of the study, oversaw the data collection, and drafted the manuscript. ML and VB recorded the data and contributed to the design and analysis. RC, JF, JL and JT contributed to the design and the interpretation of findings. All authors read, commented on and approved the final manuscript.

## Authors' information

The study took place in the context of a broader programme of research into personal recovery, described at researchintorecovery.com.

## Pre-publication history

The pre-publication history for this paper can be accessed here:

http://www.biomedcentral.com/1472-6963/10/323/prepub
